# *Andrographis paniculata* Leaf Extract Prevents Thioacetamide-Induced Liver Cirrhosis in Rats

**DOI:** 10.1371/journal.pone.0109424

**Published:** 2014-10-03

**Authors:** Daleya Abdulaziz Bardi, Mohammed Farouq Halabi, Pouya Hassandarvish, Elham Rouhollahi, Mohammadjavad Paydar, Soheil Zorofchian Moghadamtousi, Nahla Saeed Al-Wajeeh, Abdulwali Ablat, Nor Azizan Abdullah, Mahmood Ameen Abdulla

**Affiliations:** 1 Department of Biomedical Science, Faculty of Medicine, University of Malaya, Kuala Lumpur, Malaysia; 2 Department of Medical Microbiology, Faculty of Medicine, University of Malaya, Kuala Lumpur, Malaysia; 3 Department of Pharmacology, Faculty of Medicine, University of Malaya, Kuala Lumpur, Malaysia; 4 Biomolecular Research Group, Biochemistry Program, Institute of Biological Sciences, Faculty of Science, University of Malaya, Kuala Lumpur, Malaysia; 5 Institute of Biological Science, Faculty of Science, University of Malaya, Kuala Lumpur, Malaysia; Wayne State University School of Medicine, United States of America

## Abstract

This study investigated the hepatoprotective effects of ethanolic *Andrographis paniculata* leaf extract (ELAP) on thioacetamide-induced hepatotoxicity in rats. An acute toxicity study proved that ELAP is not toxic in rats. To examine the effects of ELAP *in* *vivo*, male *Sprague Dawley* rats were given intraperitoneal injections of vehicle 10% Tween-20, 5 mL/kg (normal control) or 200 mg/kg TAA thioacetamide (to induce liver cirrhosis) three times per week. Three additional groups were treated with thioacetamide plus daily oral silymarin (50 mg/kg) or ELAP (250 or 500 mg/kg). Liver injury was assessed using biochemical tests, macroscopic and microscopic tissue analysis, histopathology, and immunohistochemistry. In addition, HepG2 and WRL-68 cells were treated *in* *vitro* with ELAP fractions to test cytotoxicity. Rats treated with ELAP exhibited significantly lower liver/body weight ratios and smoother, more normal liver surfaces compared with the cirrhosis group. Histopathology using Hematoxylin and Eosin along with Masson’s Trichrome stain showed minimal disruption of hepatic cellular structure, minor fibrotic septa, a low degree of lymphocyte infiltration, and minimal collagen deposition after ELAP treatment. Immunohistochemistry indicated that ELAP induced down regulation of proliferating cell nuclear antigen. Also, hepatic antioxidant enzymes and oxidative stress parameters in ELAP-treated rats were comparable to silymarin-treated rats. ELAP administration reduced levels of altered serum liver biomarkers. ELAP fractions were non-cytotoxic to WRL-68 cells, but possessed anti-proliferative activity on HepG2 cells, which was confirmed by a significant elevation of lactate dehydrogenase, reactive oxygen species, cell membrane permeability, cytochrome *c*, and caspase-8,-9, and, -3/7 activity in HepG2 cells. A reduction of mitochondrial membrane potential was also detected in ELAP-treated HepG2 cells. The hepatoprotective effect of 500 mg/kg of ELAP is proposed to result from the reduction of thioacetamide-induced toxicity, normalizing reactive oxygen species levels, inhibiting cellular proliferation, and inducing apoptosis in HepG2 cells.

## Introduction

The liver is the largest internal organ in vertebrates, including humans. It is vital to survival, as it is the key organ of metabolism and excretion, and supports every other organ. It is continuously exposed to xenobiotics, and hence is prone to many diseases [1]. In chronically damaged liver, hepatic fibrosis arises from perpetual wound-healing, which results in abnormal production and accumulation of connective tissue and ultimately leads to liver cirrhosis [2]. The rate of fibrosis progression is reported to depend on the cause of liver disease, host, and environmental factors. Liver cirrhosis has a high global prevalence. It is the end-stage of most liver pathologies and leads to chronic liver dysfunction, altered metabolism, and death [1]. Reactive oxygen species (ROS) play an important role in liver pathology, particularly in cases of alcohol- and toxicity-induced liver disease [3]. Because conventional and synthetic drugs used to treat liver disease are often inadequate and can have serious side effects, many people, even in developed countries, turn to complementary and alternative medicine (CAM). Medicinal plants reported to possess hepatoprotective activity include *Vitex negundo* [4], *Boesenbergia rotunda* [5], *Phyllanthus niruri* [6], *Ipomoea aquatic* [7], *Orthosiphon stamineus* [8], *Zingiber officinale* [9], and *Caesalpinia sappan* [10].

*Andrographis paniculata* (Malay: Hempedu Bumi) is a herbaceous annual belonging to the family Acanthaceae, native to Southeast Asia, especially China, India, and Sri Lanka. This herb has been traditionally used in Ayurvedic medicine (Indian traditional medicine). It is most used to treat and prevent infectious diseases, as it thought to strengthen the immune system [11]. In addition, *A. paniculata* has analgesic [12], antioxidant [13], antibiofilm [14], gastroprotective [15], wound-healing [16], antiflarial [17], antimicrobial [18], [19], anticancer [20], and antimalarial effects [21]. Phytochemical analyses have revealed that it is a rich source of diterpenoids and 2′-oxygenated flavonoids, including andrographolide, neoandrographolide, 14-deoxy-11, 12-didehydroandrographolide, 14-deoxyandrographolide, isoandrographolide, 14-deoxyandrographolide-19-β-d-glucoside, homoandrographolide, andrographan, andrographosterin, and stigmasterol [22]. Andrographolide is the primary bioactive phytochemical of *A. paniculata* [23], where it is found in the leaves with a concentration of >2% [22]. When ingested orally, andrographolide accumulates in the visceral organs such as liver. It is absorbed quickly and P-glycoprotein was suggested to be participants in the intestinal absorption. Andrographolide metabolized extensively in rats and humans, structural illucidation of metabolites have shown andrographolid analogues, sulfonates and sulfate ester compounds which isolated from rat feces, urine and small intestine, achieving ∼90% elimination within 48 h [22], [24]. Andrographolide exhibited significant cytotoxic activity against cancer cells, induces apoptosis, possess anti-inflammatory and Anti-angiogenic activity and has chemo-protective potential towards normal cells [24].

Thioacetamide (TAA) is hepatotoxic, with a single dose able to produce centrilobular necrosis followed by a regenerative response in animals [25], [26]. High dosages lead to liver cirrhosis and hepatocarcinoma [27]. The aim of the present study was to evaluate the possible hepatoprotective effects of *A. paniculata* leaf extract (ELAP) both *in* *vivo* (using TAA-induced liver cirrhosis in the rat as a model system) and *in* *vitro* (using cultured hepatic cell lines). Results were compared to the effects of silymarin, a drug commonly used as a liver support during treatment of liver cirrhosis.

## Materials and Methods

### Ethics Statement

The study was approved by the Ethics Committee for Animal Experimentation, Faculty of Medicine, University of Malaya, Malaysia (Ethic No. PM/05/08/2012/MMA (a) (R)). All animals received humane care according to the criteria outlined in the Guide for the Care and Use of Laboratory Animals prepared by the United States National Academy of Sciences and published by the National Institutes of Health [28].

### Chemicals and Consumables

TAA was purchased from Sigma-Aldrich (Germany) and silymarin was from International Laboratory (USA). HepG2 human hepatocarcinoma cells and WRL-68 normal human liver cells were purchased from the American Type Culture Collection (ATCC, Manassas, USA) and cultured in supplemented Dulbecco’s modified Eagle medium (DMEM, Invitrogen, Carlsbad, CA) at 37°C in a humidified atmosphere of carbon dioxide and air (5∶95). DMEM was supplemented with 10% heat-inactivated fetal bovine serum (FBS), 100 mg/mL streptomycin, and 100 U/mL penicillin (all from Invitrogen). The MTT assay [3-(4,5-dimethylthiazol-2-yl)-2,5-diphenyltetrazolium bromide] was purchased from Invitrogen.

### Preparation of Plant Extracts (ELAP)

Air-dried *A. paniculata* leaves were obtained from Ethno Resources (Selangor, Malaysia), and their identity was confirmed by comparison with voucher specimen no. 43261 deposited at the Herbarium of Rimba Ilmu, Institute of Science Biology, University of Malaya, Kuala Lumpur. Leaves were ground to a fine powder using an electric blender, and 100 g powder was suspended in 500 mL of 95% ethanol for 3 days. This mixture was filtered using fine muslin cloth followed by Whatman no. 1 filter paper, then concentrated under reduced pressure (Eyela rotary evaporator, Sigma-Aldrich, USA), frozen, and lyophilized (yield: 4.7 g crude extract). For *in* *vivo* experiments, extract was dissolved in 10% Tween-20 and administered orally to rats at 250 or 500 mg/kg body weight (5 mL/kg body weight). For *in* *vitro* experiments, liquid-liquid partitioning was performed on crude extract to yield *n*-hexane (HF), chloroform (CF), butanol (BF), and aqueous (AF) soluble ELAP fractions [9], which were stored at −20°C.

### Acute Toxicity Test

The study was carried out using the “fix dose” method of the Organisation for Economic Co-operation and Development guideline no. 420 [29] and following the Animal Research: Reporting *In Vivo* Experiments (ARRIVE) guidelines [24]. Healthy adult female *Sprague Dawley* (SD) rats (6–8 weeks old, 180–200 g) obtained from the Animal House (Faculty of Medicine, University of Malaya) were randomly divided into two groups of 6 rats each. The treatment group received 2500 mg/kg ELAP, and the control group received only vehicle (10% Tween-20). To eliminate food from the gastrointestinal tract that might intervene with ELAP absorption, food was withheld overnight prior to ELAP administration and for another 3 to 4 h after administration. Water was provided throughout. Animals were observed for 0.5, 2, 4, 24, and 48 h for the onset of behavioral changes, clinical or toxicological symptoms, and death. Mortality was further observed over a period of 2 weeks every morning and animals were sacrificed on day 15. Gross necropsies were performed, histopathology was performed on livers and kidneys, and serum biochemistry was assessed following standard methods [30].

### In Vivo Experimental Design

Healthy adult male SD rats (6–8 weeks old, 150–180 g) were obtained from the Animal House. Rats were housed individually in cages with wide-mesh wire bottoms in an animal room at 25°*±*2°C, 50–60% humidity, and exposed to a 12-h light/dark cycle. Animals were maintained on standard pellet diet and tap water. Animals were acclimatized under standard laboratory conditions for a period of 2 weeks before the experiment.

Rats were divided randomly into five groups of six rats each. The normal control group was injected intraperitoneally with vehicle (10% Tween-20, 5 mL/kg) thrice weekly and received daily oral of distilled water (5 mL/kg). A TAA control group (to establish the liver cirrhosis model) was injected intraperitoneally with 200 mg/kg TAA thrice weekly for eight weeks [31] and received daily oral administration of vehicle (10% Tween-20, 5 mL/kg). A positive control group was injected intraperitoneally with 200 mg/kg TAA thrice weekly and given daily oral silymarin at 50 mg/kg. The two experimental groups were injected intraperitoneally with 200 mg/kg TAA thrice weekly and given daily oral ELAP at 250 or 500 mg/kg.

The experiment was carried out for 8 weeks [5]. The animals were given water ad libitum. The body weights of the animals were recorded weekly. At the end of the 8^th^ week, rats were fasted for 24 h after the last treatment and then anesthetized under ketamine (30 mg/kg, 100 mg/mL) and xylazil (3 mg/kg, 100 mg/mL) anesthesia. Blood was withdrawn through the jugular vein and collected in gel-activated tubes, which were allowed to clot, centrifuged, and analyzed at the Clinical Diagnosis Laboratory of the University of Malaya Hospital for liver function test.

### Macroscopic Liver Assessment

The abdominal and thoracic cavities were opened and rats displaying macroscopic evidence of pathology in organs other than the liver were excluded from the study. The livers were weighed and carefully examined for any gross pathology, then washed in ice-cold saline, blotted on filter paper, and weighed. Histopathology and immunohistochemistry was performed on liver tissues. Additionally, antioxidant activity and levels of oxidative stress were measured.

### Histopathology

Liver specimens were fixed in 10% buffered formalin and processed by an automated tissue processing machine (Leica, Germany). Sections were stained with hematoxylin and eosin (H&E) and Masson’s trichrome. Stained liver slices were evaluated under a Nikon microscope (Y-THS, Japan).

### Immunohistochemistry

Liver tissue sections were heated at 60*°*C for 30–60 min in an oven (Venticell, MMM, Einrichtungen, Germany) and deparaffinized in xylene (2×3 min). Tissues were rehydrated using absolute, 95%, and 70% alcohol (2×3 min each) followed by running water. Antigen retrieval was performed in 10 mM sodium citrate buffer boiled in a microwave (Sanyo, Super Showe wave, Japan) for 10 min. The tissue slides were then cooled and placed in Tris Buffered Saline (TBS) with 0.05% Tween-20. Immunohistochemistry was performed following manufacturer’s instructions using ARK (Animal Research Kit), Peroxidase (kit #K3955) (DakoCytomation, USA). Endogenous peroxidase was quenched using peroxidase blocking solution (0.03% hydrogen peroxide sodium azide) for 5 min. The slides were rinsed gently with distilled water for 3 min and then incubated with biotinylated primary antibodies against (PCNA; 1∶200) for 15 min. Tissue sections were gently washed twice with wash distilled water 3 min and kept in the buffer bath in a humid chamber. Streptavidin-HRP was added and sections were incubated for 15 min, then washed twice with distilled water for 3 min. Tissue sections were incubated with diaminobenzidine (DAB) substrate Chromagen for 5 min, then washed and counterstained with hematoxylin for 5 sec. Finally, sections were dipped ten times in weak ammonia (0.037 M/L), rinsed in distilled or deionized water for 2 to 5 min, and mounted with mounting medium. Under a light microscope, positive antigens stained brown against a blue hematoxylin background. The proliferation index of PCNA-stained liver sections was assessed by counting the percentage of labeled cells per 1000 liver cells, and the number of mitotic cells was expressed as mitotic index [9].

### Antioxidant Activity and Oxidative Stress

Liver samples were washed, placed in ice-cold 10% (w/v) phosphate buffer solution (PBS), pH 7.4, and then homogenized on ice using a Teflon homogenizer (Polytron, Heidolph RZR 1, Germany). Cell debris was removed by centrifugation at 4500 rpm for 15 min at 4*°*C. The supernatant was used to determine antioxidant activity using assay kits for superoxide dismutase and catalase (SOD and CAT; Cayman Chemical Company, USA), and to analyze oxidative stress using assay kits for thiobarbituric acid reactive substance (TBARS; Cayman Chemical Company) and nitric oxide (QuantiChrom, USA).

### Biochemical Parameters

Blood was collected into clot-activator tubes and serum was separated by centrifuging at 2500 rpm for 15 min. Aspartate aminotransferase (AST), alanine aminotransferase (ALT), total protein, albumin, globulin, total bilirubin, conjugated bilirubin, alkaline phosphatase (ALP), and gamma glutamyl transferase (GGT) were assayed spectrophotometrically at Clinical Diagnostic Laboratory of the University Malaya Medical Centre.

### *In Vitro* Cell Viability Assay

Cell viability was measured using a standard colorimetric MTT reduction assay. HepG2 and WRL-68 cells were cultured in 96-wells plates at a density of 5×10^5^ cells/well. After 24 h, cells were treated with increasing concentrations of the four ELAP fractions (CF, HF, BF, and AF) or 0.2% DMSO (vehicle control) for 24 h. Next, 2 mg/mL MTT (Invitrogen) was added and the cells were incubated for 4 h. After incubation, the supernatants were aspirated, and 100 µL DMSO were added to dissolve formazan crystals. Optical density was measured at 570 nm using a multiwell plate reader (Asys UVM340, Eugendorf, Austria) and IC_50_ values of the isolated fractions were determined from the linear portion of dose-response curves. Three ELAP fractions (CF, HF, and BF) demonstrated significant antiproliferative effects in HepG2 cells and were used for further investigation.

### Lactate Dehydrogenase (LDH) Release Assay

To confirm the cytotoxicity of the three ELAP fractions, we measured LDH released from cells into the medium using a Pierce LDH cytotoxicity assay kit (Thermo Scientific, Pittsburgh, PA). HepG2 cells were incubated with different concentrations of isolated ELAP fractions for 48 h and then LDH activity was determined in the supernatant. As a positive control, cells were completely lysed using Triton X-100 (2%) to give maximum LDH release. LDH reaction solution (100 µL) was added to the supernatants **(**100 µL) for 30 min and red color intensity measured at 490 nm. The level of LDH released from treated cells was expressed as a percentage of the positive control.

### Reactive Oxygen Species (ROS) Assay

To determine the effect of ELAP fractions on ROS formation in HepG2 cells, 1×10^4^ HepG2 cells per well were seeded in 96-well plates and incubated at 37°C for 24 h. Cells were then treated with ELAP fractions at different concentrations for 24 h. Treated cells were stained with 50 µL dihydroethidium (2.5 µg/mL) for 30 min and then washed twice with PBS. ROS formation was measured at 490 nm using a fluorescence microplate reader (Tecan Infinite M 200 PRO, Männedorf, Switzerland).

### Multiple Cytotoxicity Assay

A Cellomics high-content screening (HCS) system and multiparameter Cytotoxicity 3 kit (Thermo Scientific) were used to simultaneously analyze cell membrane permeability, cytochrome *c* release, and mitochondrial membrane potential (MMP) in ELAP-treated HepG2 cells. Briefly, 1×10^4^ liver cancer HepG2 cells were seeded into 96-well plates and incubated at 37°C for 24 h. Cells were then incubated with different concentrations of ELAP fractions for 24 h. Treated cells were stained with cell permeability and MMP dyes, fixed with 3.5% paraformaldehyde, blocked with 1×blocking buffer, and labeled by cytochrome *c* immunohistochemistry according to the vendor's protocol. Hoechst 33342 dye was used to counterstain the nucleus and cells were analyzed using the ArrayScan HCS system with ArrayScan II Data Acquisition and Data Viewer version 3.0 (Cellomics).

### Caspase Activity Assay

Caspase activity in ELAP-treated HepG2 cells was determined using Caspase-Glo-3/7, -8, and -9 assay kits (Promega, Madison, WI). Briefly, HepG2 cells (1×10^4^ cells/well) were cultured in a white 96-well microplate overnight. Cells were treated with different concentrations of isolated ELAP fractions for 24 h. Next, Caspase-Glo reagent (100 µL) was added, and cells were incubated for 30 min. Caspase activities were measured by luminescence measurement using a microplate reader (Infinite M200 PRO Tecan, Austria).

### Statistical Analysis

Data from the rat study were expressed as mean ± SEM using one-way analysis of variance (ANOVA) followed by Bonferroni’s post hoc test using SPSS software for Windows, version 20 (SPSS, Chicago, IL, USA). *In vitro* study results were expressed as mean ± standard deviation (SD) of at least three independent experiments. ANOVA was performed using GraphPad Prism software (GraphPad Software, San Diego, CA). The level of statistical significance was set at 0.05 for all experiments.

## Results

### Acute Toxicity Analysis

Animals pretreated with 2500 mg/kg ELAP or vehicle were observed for 14 days. All animals were alive and there were no signs of toxicity-related macroscopic or behavioral abnormalities. Biochemical and histopathological analyses showed no significant difference between control and ELAP-treated groups (Figure S1 and Tables S1, S2). These findings verify that 2500 mg/kg ELAP was safe and non-toxic in rats.

### Effects of ELAP on TAA-Induced Liver Cirrhosis

#### Body Weight, Liver Weight, and Liver Index

Body weight of all animals was measured weekly and prior to scarification. Body and liver weights after two months of ELAP treatment are shown in Table 1. The control group had normal weight gains from 194 to 347 g over 8 weeks. The TAA control group weighed significantly (*P<*0.05) less than all other groups. The highest liver index (liver/body weight ratio) was observed in the TAA control group. Administration of 250 or 500 mg/kg ELAP significantly (*P<0.05*) lowered the liver index, an effect comparable to that in the silymarin administered group.

**Table 1:** Effect of ELAP on body weight, liver weight, and liver index after 8 weeks.

Treatment	Body weight (g)	Liver weight (g)	Liver index (LW/BW %)
**Normal control**	347±7	10.33±1.15	2.95±0.22
**TAA control**	172±6#	12.61±0.41#	7.38±0.36#
**Silymarin (50** **mg/kg)**	374±8*	10.66±1.69	2.99±0.32*
**ELAP (250** **mg/kg)**	236±8* #	10.92±0.59#	4.66±0.14* #
**ELAP (500** **mg/kg)**	231±4* #	10.85±0.31#	4.75±0.24* #

Data expressed as mean *±* SEM (*n = *6 rats/group).

*P<0.05 compared with TAA control,

# *P<0.05* compared with normal control.

#### Macroscopic Appearance of Liver

**Figure 1:**
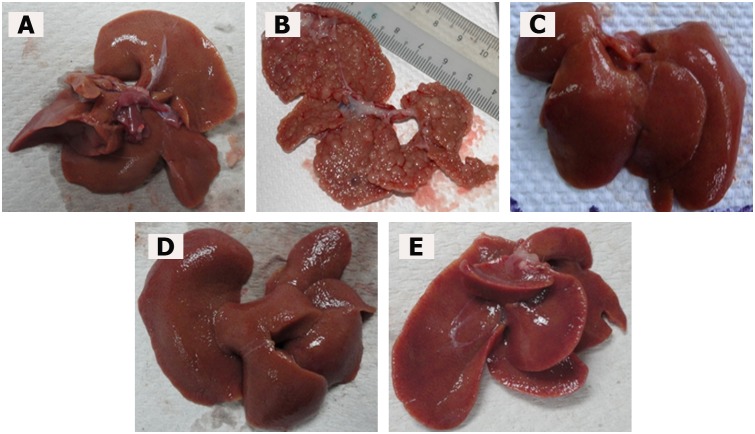
Macroscopic liver appearance. (A) Normal control liver has regular smooth surface. (B) TAA control liver has rough nodular surface, with uniform distribution of micronodules (<0.3 cm) and macronodules (≥0.3 cm). (C) TAA+silymarin liver has normal smooth surface. (D) TAA+250 mg/kg ELAP liver has nearly smooth surface and few micronodules. (E) TAA+500 mg/kg ELAP liver has normal smooth surface and nearly normal anatomical shape and appearance. Livers shown are representative samples (*n* = 6/group).

In this study, TAA treatment caused liver cirrhosis in rats. In comparison to the normal liver topology with a regular and smooth surface, the livers in the TAA control group were rough and nodular, with uniform micronodules (<0.3 cm) and macronodules (≥0.3 cm) throughout. Treatment with either silymarin or ELAP remarkably enhanced the recovery of TAA-induced liver structure damage as shown in Figure 1.

#### Histopathological Analysis of Liver Sections

**Figure 2:**
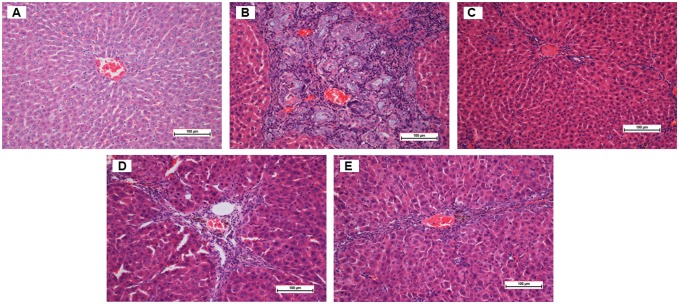
H&E staining of histopathological liver sections. (A) Normal control liver has normal histological structure and architecture. (B) TAA control liver has structural damage, irregular regenerating pseudolobules with dense fibrotic septa, proliferation of bile duct, and presence of centrilobular and inflammatory cells. (C) TAA+silymarin liver has mild inflammation but no fibrotic septa. (D) TAA+250 mg/kg ELAP liver has partially preserved hepatocytes, small area of necrosis, narrow fibrotic septa. (E) TAA+500 mg/kg ELAP liver has partially preserved hepatocytes and small areas of mild necrosis. Sections shown are from representative samples (*n* = 6/group).

Normal control livers were clear of any pathological abnormality, having distinct plates of hepatic cells, sinusoidal spaces, and a central vein (Figure 2A). Hepatocytes were polygonal with well-preserved cytoplasm and prominent nuclei. In contrast, TAA control livers (Figure 2B) showed a loss of normal architecture, with signs of inflammation and congestion with cytoplasmic vacuolation, fatty changes, sinusoidal dilatation, and centrilobular necrosis. The presence of regenerating micro- and macronodules, with bundles of collagen surrounding the lobules, resulted in large fibrous septa accompanied by severe proliferation of bile duct, heavy invasion of inflammatory cells, and distorted tissue architecture. Degenerative cell changes (such as cloudy swelling, hydropic degeneration, loss of nucleus and nucleolus, and necrosis) were also observed.

Rat livers treated with 250 mg/kg ELAP (Figure 2D) showed fewer macronodules and fibrotic nodules compared with TAA controls, as well as reduced inflammation and necrosis of hepatocytes with mild cytoplasmic vacuolation, and almost no visible changes compared to the reference group, with the exception of regenerative parenchyma nodules surrounded by septa of fibrous tissue. Livers from animals fed with 500 mg/kg ELAP (Figure 2E) showed remarkable histological differences compared with those treated with 250 mg/kg ELAP. They had regular hepatic architecture, minimal disruption of hepatic cellular structure, well-preserved cytoplasm, very minor fibrotic septae, and low lymphocyte infiltration. Similar effects were found in silymarin-treated rats (Figure 2C). These results indicate that 500 mg/kg ELAP administration was as effective as silymarin in protecting rat liver against cirrhosis.

#### Masson’s Trichrome Staining of Liver Sections

**Figure 3:**
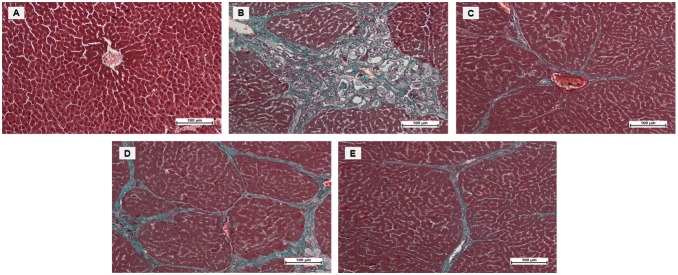
Masson’s Trichrome staining of histopathological liver sections. (A) Normal control liver has normal architecture. (B) TAA control liver shows proliferation of bile duct, dense fibrous septa, and collagen fibers. (C) TAA+silymarin liver shows minimal fibrous septa and collagen fibers. (D) TAA+250 mg/kg ELAP liver shows moderate fibrous septa and irregular regenerating nodules. (E) TAA+500 mg/kg ELAP liver shows mild fibrous septa and collagen fibers. [Sections shown are from representative samples (*n* = 6/group)].

Masson’s Trichrome staining of control and ELAP-treated liver sections is shown in Figure 3. Liver tissues from normal controls showed no collagen deposition, whereas those from TAA controls showed bile duct proliferation with dense fibrous septa and increased deposition of collagen fibers around the congested central vein, indicating severe fibrosis. Liver tissues from silymarin-treated rats showed minimal collagen deposition, indicating minimal fibrosis. Livers from rats treated with 250 mg/kg ELAP showed moderate deposition of collagen fibers and moderate congestion around the central vein, and those from rats treated with 500 mg/kg ELAP showed only mild collagen deposition and mild congestion around the central vein. This supports the protective effects of ELAP against TAA-induced hepatic toxicity.

#### Immunohistochemical Staining of Liver Sections

**Figure 4:**
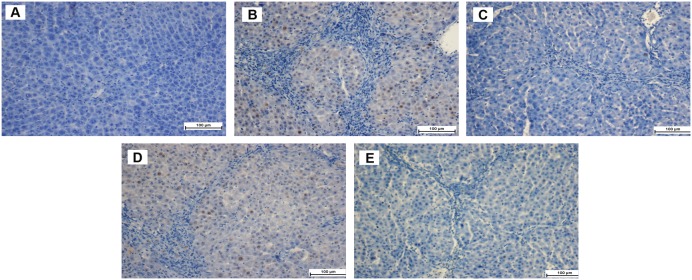
PCNA labeling in histopathological liver sections. (A) Normal controls stained without primary antibody show normal liver architecture and no PCNA labeling. (B) TAA controls have many PCNA-positive hepatocyte nuclei. (C) TAA+silymarin rats have no PCNA-positive hepatocytes. (D) TAA+250 mg/kg ELAP rats have moderate hepatocyte regeneration, as indicated by moderate presence of PCNA-positive hepatocyte nuclei. (E) TAA+500 mg/kg ELAP rats have mild PCNA expression with few regenerative hepatocytes.

The effect of ELAP on cell proliferation following TAA-induced liver damage was examined by immunohistochemical analysis of PCNA expression in the liver parenchyma using anti-PCNA antibody (Figure 4 and Table 2). Hepatocytes of the normal control group showed no PCNA staining, indicating that no cell regeneration was occurring. In contrast, hepatocytes of the TAA control group had upregulated PCNA expression and an elevated mitotic index, indicating proliferation to repair the severe liver tissue damage induced by TAA. Liver tissues treated with 250 mg/kg ELAP, 500 mg/kg ELAP, or silymarin had reduced hepatocyte regeneration compared to the TAA controls, as indicated by reduced PCNA expression and a significant reduction of the mitotic index. ELAP had an outstanding effect on PCNA labeling and mitotic index in a dose-dependent manner.

**Table 2:** Effect of ELAP on PCNA labeling and mitotic index.

Treatment	PCNA labeling	Mitotic index^a^
**Normal control**	0	0
**TAA control**	24.83±0.92#	72.5±1.22#
**Silymarin (50** **mg/kg)**	0.33±0.19* #	16.83±1.10* #
**ELAP (250** **mg/kg)**	11.66±1.64* #	42.33±1.28* #
**ELAP (500** **mg/kg)**	0.66±0.19* #	20.83±1.53* #

a Percentage of labeled cells per 1000 liver cells. Data expressed as mean *±* SEM (*6* =  rats/group).

*P<0.05 compared with TAA control,

# *P<0.05* compared with normal control.

#### Effect of ELAP on Liver Antioxidant Enzyme Levels

**Figure 5:**
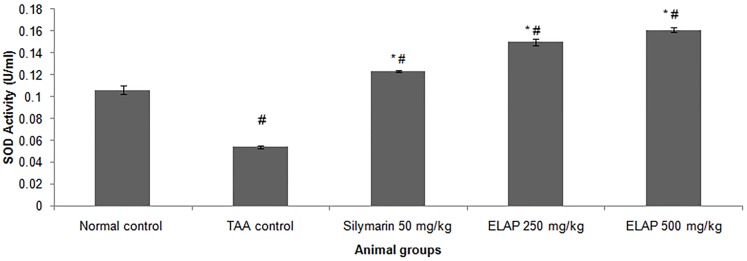
Effect of ELAP on SOD activity in liver tissue. Data expressed as mean *±* SEM (*n = *6 rats/group). *P<0.05 compared with TAA control, *^#^P<0.05* compared with normal control.

**Figure 6:**
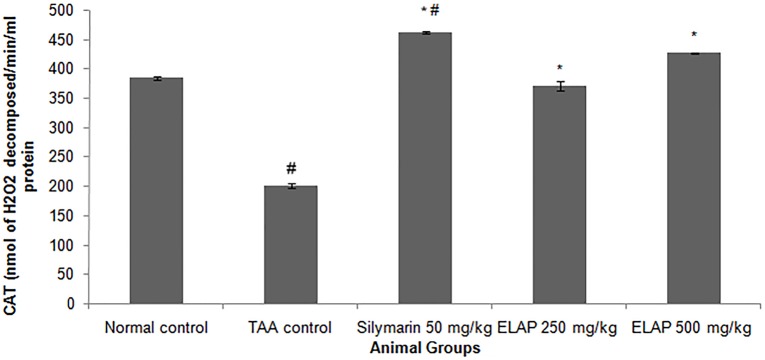
Effect of ELAP on CAT activity in liver tissue. Data expressed as mean *±* SEM (*n = *6 rats/group). *P<0.05 compared with TAA control, *^#^P<0.05* compared with normal control.

Antioxidant enzymatic activities of SOD and CAT in liver homogenates are shown in Figures 5 and 6. SOD activity was decreased in TAA controls compared with normal controls (0.054±0.00 vs. 0.106±0.01 U/mL SOD, respectively). SOD activity was significantly higher in ELAP-treated groups (0.149±0.01 and 0.161±0.01 U/mL SOD for 250 and 500 mg/kg ELAP, respectively) compared with TAA controls (P<0.05 for each), and was comparable to the silymarin-treated group (0.123±0.01 U/mL SOD). Similarly, CAT activity was reduced in TAA controls compared with normal controls (200±2.98 vs. 384.00±1.93 nmol H_2_O_2_ decomposed/min/mL protein, respectively). As for SOD, CAT activity in the ELAP-treated groups was significantly higher than in TAA controls (370.42±5.50 and 426.24±9.92 nmol H_2_O_2_ decomposed/min/mL protein for 250 and 500 mg/kg ELAP, respectively; P<0.05 for each), and comparable results were found in the silymarin-treated group (472.01±5.80 nmol H_2_O_2_ decomposed/min/mL protein).

#### Effect of ELAP on Oxidative Stress

**Figure 7:**
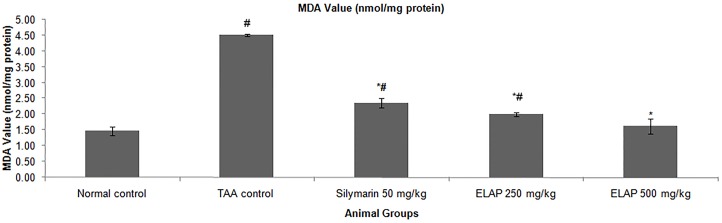
Effect of ELAP on MDA levels in liver tissue. Data expressed as mean *±* SEM (*n = *6 rats/group). *P<0.05 compared with TAA control, *^#^P<0.05* compared with normal control.

**Figure 8:**
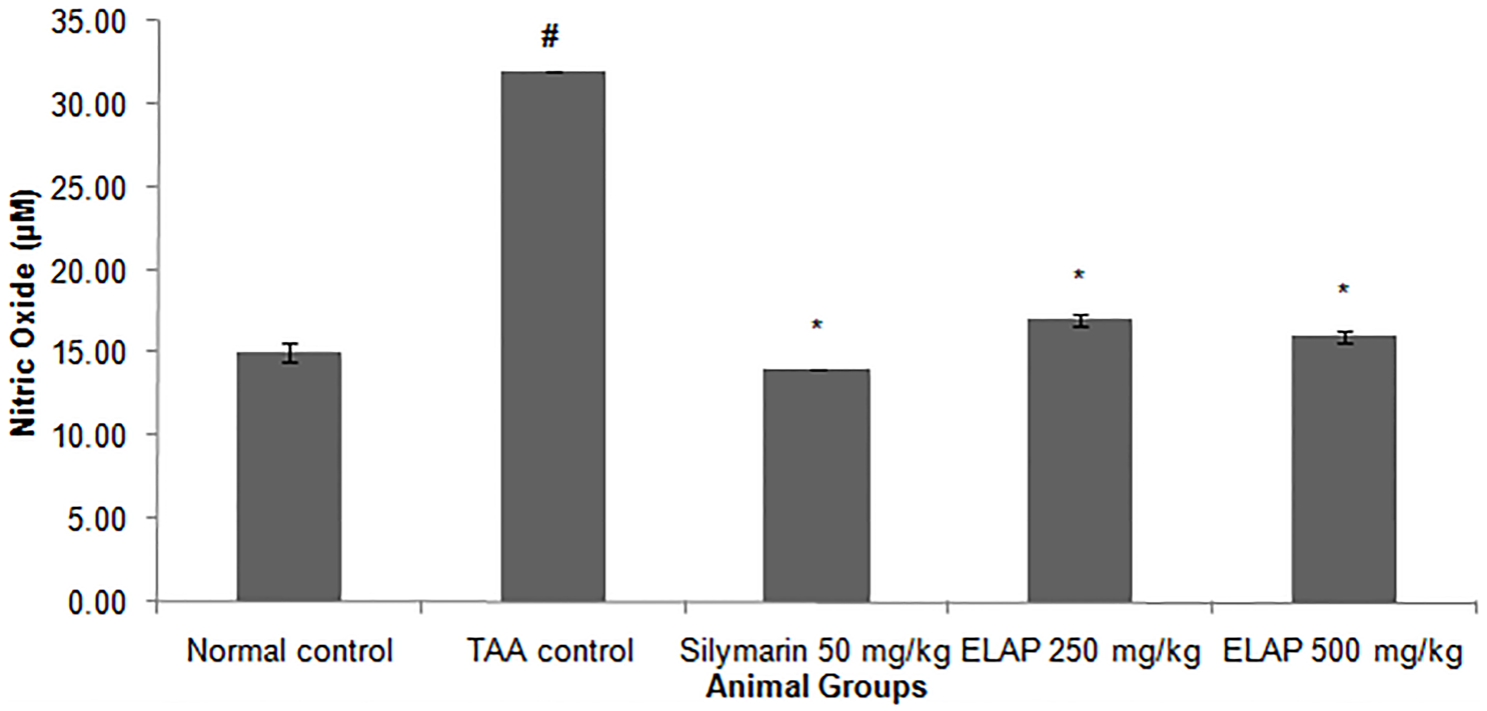
Effect of ELAP on NO levels in liver tissue. Data expressed as mean *±* SEM (*n = *6 rats/group). *P<0.05 compared with TAA control, *^#^P<0.05* compared with normal control.

Oxidative stress in liver tissue was assessed by measuring MDA and NO levels in liver homogenates, as shown in Figures 7 and 8. MDA levels increased significantly in TAA-treated rats compared with normal control rats (4.52±0.01 vs. 1.47±0.11 nmol/mg protein, respectively; *P<*0.05). Compared to TAA-treated animals, administration of 500 mg/kg ELAP significantly lowered the MDA level (1.62±0.00 nmol/mg protein; *P<0.05*), restoring a level comparable to normal controls. NO levels, which indicate severe cell damage in cirrhotic livers, also increased significantly in TAA-treated rats (32.00±0.68 µM) compared with the other groups (*P<0.05*). ELAP significantly restored the altered NO level (17.00±0.42 and 16.00±0.93 µM for 250 and 500 mg/kg ELAP, respectively; *P<0.05* for each), and the same effect was found in the silymarin group (14.00±0.48 µM).

#### Effect of ELAP on Biochemical Parameters

Administration of TAA led to significant increases of ALT, AST, ALP, globulin, total bilirubin, conjugated bilirubin, and GGT, which are indicators of liver injury (Tables 3 and 4). Significant decreases in total protein and albumin were also observed, indicating acute hepatocellular damage. ELAP and silymarin treatment significantly reduced enzyme activities of ALT, AST, ALP, globulin, total bilirubin, conjugated bilirubin, and GGT. Additionally, total protein and albumin levels were increased in ELAP- or silymarin-treated groups compared with the TAA controls. Thus, ELAP counteracted the toxic effect of TAA by restoring normal liver function. A marginal effect was observed at a dose of 250 mg/kg, whereas ELAP effectively prevented TAA-induced liver damage at a dose of 500 mg/kg.

**Table 3:** Effect of ELAP on biochemical parameters in TAA-induced liver cirrhosis.

Treatment	Total protein (g/L)	Albumin (g/L)	Globulin (g/L)	Total bilirubin (µM)	Conjugated bilirubin (µM)
**Normal control**	68.67±1.4	12.83±0.70	54.5±0.22	2.7±0.21	1±0.00
**TAA control**	60.83±0.47#	7.83±0.16#	68.66±1.4#	9±0.21#	5.3±0.00#
**Silymarin (50 mg/kg)**	67.33±0.95*	11.83±0.74*	54.33±1.47*	5.6±0.76	3±0.36* #
**ELAP (250 mg/kg)**	61.5±0.67* #	11.66±0.21*	48±0.85* #	7±0.36* #	3.5±0.61* #
**ELAP (500 mg/kg)**	70±1.46*	12.16±0.30*	54.5±0.5*	5±0.77* #	3±0.22* #

Data expressed as mean *±* SEM (*n = *6 rats/group).

*P<0.05 compared with TAA control,

# *P<0.05* compared with normal control.

**Table 4:** Effect of ELAP on serum liver biomarkers in TAA-induced liver cirrhosis.

Treatment	ALP (IU/L)	ALT (IU/L)	AST (IU/L)	GGT (IU/L)
**Normal control**	100.66±2.02	64±0.516	174.5±3.31	5±0.00
**TAA control**	243.83±7.44#	209.83±2.13#	322.16±2.52#	12±0.00#
**Silymarin (50 mg/kg)**	132.66±10.44* #	70.3±2.01*	184.33±3.25*	7±0.00*
**ELAP (250 mg/kg)**	179.5±2.34* #	76.5±7.45*	246.5±31.7	6.83±0.16* #
**ELAP 500 mg/kg**	125.5±2.18* #	52.67±4.65*	190.67±21.7*	6.67±0.55*

Data expressed as mean *±* SEM (*n = *6 rats/group).

*P<0.05 compared with TAA control,

# *P<0.05* compared with normal control.

### *In Vitro* Evaluation of ELAP Fractions

#### Effect of ELAP fractions on HepG2 Cancer Cells Proliferation

To determine the anti-proliferative effect of the four isolated ELAP fractions in HepG2 and WRL-68 cells, the MTT assay was carried out. CF, HF, and BF had a significant anti-proliferative effect on HepG2 cells (Table 5). CF had the strongest cytotoxic effect, with an IC_50_ value of 17.43±0.93****µg/mL after 24****h, whereas AF did not affect HepG2 cell proliferation. Normal human hepatic WRL-68 cells were not affected by treatment with any of the four fractions, indicating a selective cytotoxicity in HepG2 cells.

**Table 5:** IC_50_ values of isolated fractions on HepG2 and WRL-68 cells after 24****h.

	IC_50_ (µg/mL)			
Cell line	CF	BF	HF	AF
**HepG2**	17.43±0.93	19.541±1.113	22.787±1.812	>100
**WRL-68**	52.124±1.356	58.853±2.45	61.43±2.88	>100

Data expressed as mean ± standard deviation (SD) of at least three independent experiments.

#### Effect of ELAP fractions on LDH Leakage

**Figure 9:**
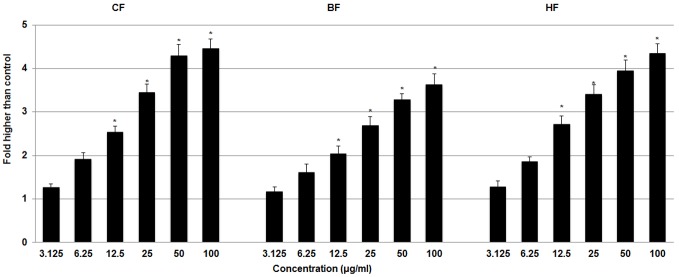
Effect of ELAP fractions on LDH leakage in HepG2 cells. LDH assay was used to assess the loss of membrane integrity in HepG2 cells treated with CF, HF, or BF. Significant cytotoxicity was observed at 12.5 to 100 µg/mL. Data represent mean ± SD of three independent experiments. *P<0.05 compared with no treatment.

To test the effects of ELAP fractions on membrane integrity, we measured the levels of LDH released into the medium of HepG2 cells following treatment with CF, HF, or BF. CF, HF, and BF significantly increased LDH release in a dose-dependent manner at concentrations of 12.5 to 100****µg/mL (Figure 9).

#### Effect of ELAP fractions on ROS Generation

**Figure 10:**
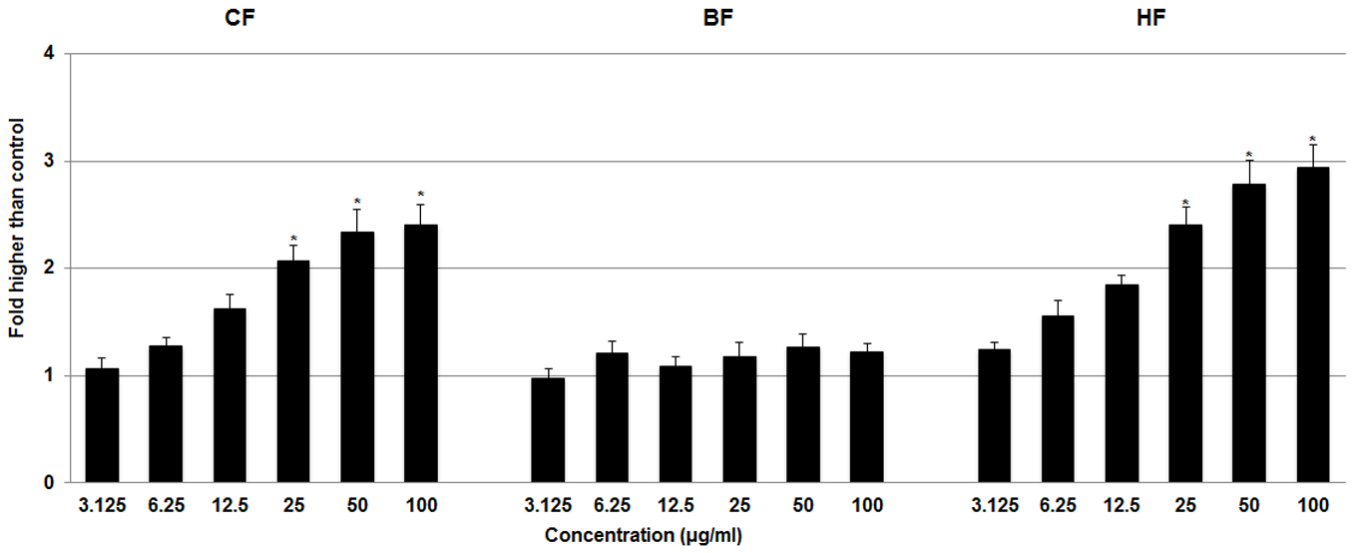
Effect of ELAP fractions on ROS generation in HepG2 cells. The level of ROS increased significantly after CF and HF treatment at concentrations from 25 to 100 µg/mL. Data represent mean ± SD of three independent experiments. *P<0.05 compared with no treatment.

ROS generation can lead to metabolic impairment and cell death. We measured the formation of ROS in HepG2 cells after treatment with CF, HF, and BF. We found a significant dose-dependent generation of ROS in HepG2 cells treated with CF and HF at 25 to 100 µg/mL, whereas BF did not cause ROS generation (Figure 10).

#### Effect of ELAP fractions on Mitochondria-Initiated Events

**Figure 11:**
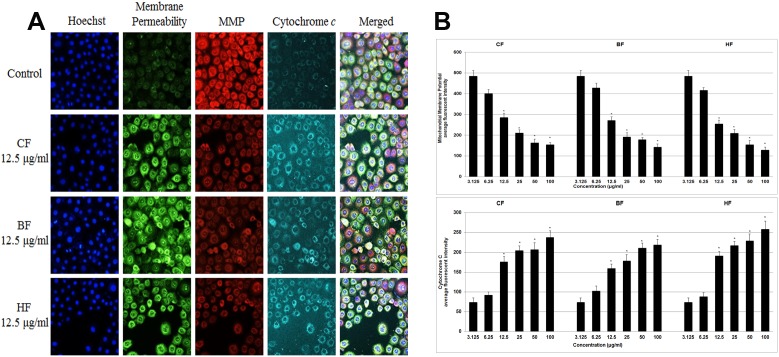
Effect of ELAP fractions on apoptotic markers in HepG2 cells. (A) HepG2 cells were treated with medium alone and 12.5 µg/mL CF, HF, or BF, then stained with Hoechst 33342, cell membrane permeability, MMP, and cytochrome *c* dyes. Isolated fractions caused a marked elevation in cytochrome *c* release and cell membrane permeability, and a noticeable decrease in MMP. (B) Dose-dependent reduction of MMP and increase in cell permeability in treated HepG2 cells (12.5 to 100 µg/mL). Cytochrome *c* was significantly released at CF, HF, or BF concentrations of 12.5 to 100 µg/mL. Data represent mean ± SD of three independent experiments. *P<0.05 compared with no treatment.

To investigate the effects of fractions on apoptosis, multiple parameters were measured in treated and control HepG2 cells. Untreated HepG2 cells were strongly stained with MMP dye, whereas cells treated for 24****h with CF, HF, or BF (12.5 to 100 µg/mL) were not (Figure 11). Also, cytochrome *c* was released from mitochondria at these concentrations, and cell membrane permeability was significantly elevated (Figure 11).

#### Effect of ELAP fractions on Caspase Activation

**Figure 12:**
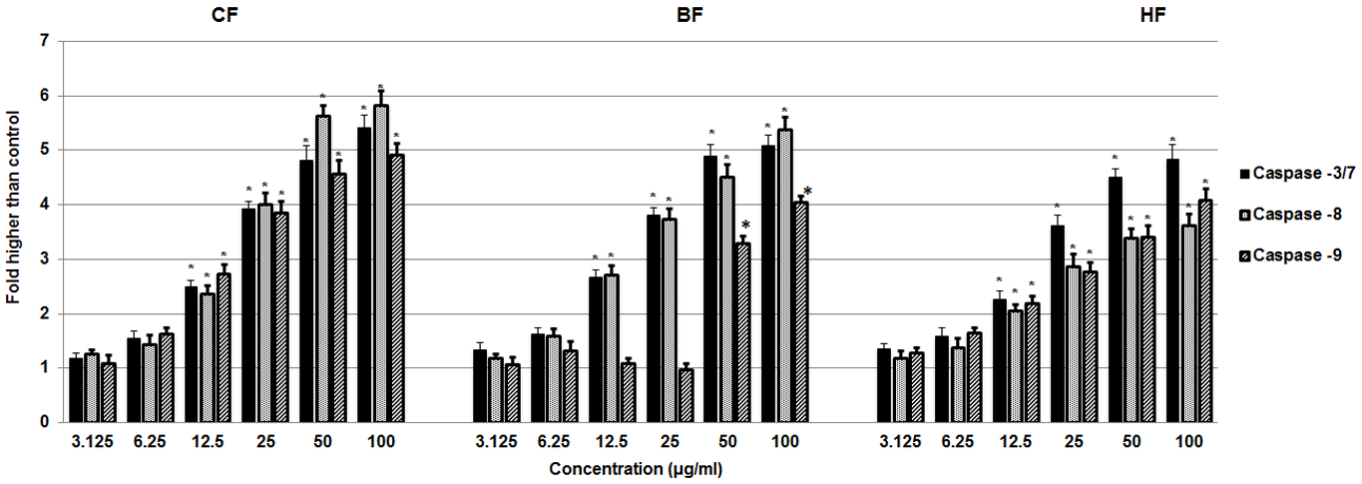
Effect of ELAP fractions on Caspases activation in HepG2 cells. Relative luminescence dose-dependent expression of caspase-3/7, -8, and -9 in HepG2 cells treated with CF and HF caused significant activation at 12.5 to 100 µg/mL concentrations. In HepG2 cells treated with CF and HF, caspase-3/7, -8, and -9 expression increased significantly and in a dose-dependent manner at concentrations from 12.5 to 100 µg/mL. BF activated caspase-3/7 and -8 at the same concentrations, but caspase-9 was activated only at higher concentrations (50 and 100 µg/mL). Activation was measured by relative luminescence**.** Data represent mean ± SD of three independent experiments. *P<0.05 compared with no treatment.

Since caspases are key mediators of apoptotic pathways, we measured caspase activation in HepG2 cells. After 24 h treatment, CF, HF, and BF all activated caspase-3/7 and -8 at concentrations of 12.5 to 100 µg/mL caspases (Figure 12). CF and HF also activated caspase-9 at these concentrations, whereas BF activated caspase-9 only at higher concentrations (50 and 100 µg/mL). At these higher concentrations (50 and 100 µg/mL), executioner caspase-3/7 was induced all fractions more than 4-fold compared with control. High caspase-8 and -9 levels were also induced by treatment with CF, HF, and BF.

## Discussion

The liver is a sensitive organ and is more prone to toxic injuries than other organs. Several phytochemicals exhibit hepatoprotective effects in liver injury [32]; however many are toxic, which limits their clinical use [33]. We evaluated the acute toxicity of ELAP in female rats and found no toxic effects. This is in accordance with another study that reported the extract to be genotoxically safe [11]. Our observations, however, are in contrast to findings reported by Oyewo et al. that administration of *A. paniculate* aqueous extract induced chronic inflammatory responses in tissues that may caused by disruption of the plasma membrane at high doses of the extract. [34]. In this study, we observed a significance reduction in body weight in the TAA control group and ELAP-treated groups (500 mg/kg and 250 mg/kg) compared to normal control. In contrast, liver weight increased significantly in the TAA control group. ELAP treatment, however, reduced the liver weight to almost normal values. This effect might be due to reduced inflammation [35].

In this study, TAA-induced hepatotoxicity was correlated with a marked increase in serum liver biomarkers ALP, ALT, AST, and GGT. These values were significantly reduced upon ELAP administration. Similar observations of serum liver biomarkers improvement with ELAP treatment were previously reported [36]–[38]. TAA was reported to interfere with RNA movement from the nucleus to the cytoplasm, causing membrane damage that results in increased release of serum liver markers [39], [40]. In this study, TAA injection at a dose (200 mg/kg, 3 times/week caused liver cirrhosis in rats. The severe damage was ameliorated by ELAP treatment in a dose - dependent manner. These observations are in agreement with a study by Rajalakshmi et al. [41]. Blocking normal liver cells regeneration will result in hepatic fibrosis. Brenner [42] reported that telomerase, b-cell lymphoma-extra large (Bcl-xL) and adiponectin genes are sensitive to fibrosis while Fas and cathepsin B genes are resistant to hepatocyte apoptosis and, thus, are resistant to hepatic fibrosis. When hepatocytes undergo apoptosis they produce chemokines and cytokines, including macrophage inflammatory protein 2 (MIP-2), keratinocyte-derived chemokine (KC) and transforming growth factor β (TGF-β), which in turn activate hepatic stellate cells (HSCs) to lose their retinoid and express α-smooth muscle actin (α-SMA) and produce extracellular matrix (ECM) proteins. This is result in excessive production of collagens and degradation of the normal ECM [42]. Reduced collagen synthesis with ELAP administration as seen in Masson's trichrome stained slides indicates the antiapoptotic properties of ELAP, which might result from andrographolide ability to decrease α-SMA and TGF-β [43], which are implicated in apoptosis of activated hepatic stellate cells [44].

PCNA has recently been identified as the polymerase S accessory protein [45]. Ng et al. [46] reported a significant relationship between PCNA and tumor invasiveness. In this study, normal liver controls and silymarin-treated samples showed no significant PCNA staining, indicating the absence of cellular regeneration. An upregulation of PCNA expression was observed in TAA-treated controls, indicating extensive proliferation, likely in an attempt to repair TAA-induced tissue damage [5]. On the other hand, treatment using silymarin or ELAP decreased cellular proliferation levels due to a reduction in PCNA expression. Luo et al. [47] found that andrographolide is able to inhibit the expression and activity of matrix metalloproteinase (MMP)-9 by inhibiting (NF)-κB-mediated MMP-9 expression, thus preventing the proliferation of tumor cells. Decreased SOD and CAT activities in the TAA control group may be due to excessive ROS generation in cirrhotic tissue as a result of over expression of NADPH oxidase [48]. On the other hand, the increase in enzyme activities upon ELAP or silymarin treatment may stem from a decrease in ROS and free radicals, due to scavenging by andrographolides. This finding is in accordance with similar reports on the effects of *A. paniculata* on liver damage [36], . The decreased hepatic antioxidant enzyme activities of the TAA control group could explain the elevated MDA lipid peroxidation (as assessed by the TBARS assay) and the increase in NO concentration. ROS and NO are known to be involved in apoptosis of hepatocytes [49]. Administration of both ELAP and silymarin reduced MDA activity and reduced TAA-induced liver toxicity. Similar trends were observed in *A. paniculata’s* protective effect against ethanol-induced hepatotoxicity [50]. Compared with the TAA control group, both ELAP and silymarin treatment significantly reduced NO generation in liver cells. However, the presence of 14-deoxyandrographolide and 14-deoxy-11,12-didehydroandrographolide, major diterpenoids, reported to stimulate NO release in tissue endothelial cells [51]. The authors reported that 14-deoxy-11,12-didehydroandrographolide caused NO stimulation via activation of constitutive nitric oxide synthase (cNOS), which was followed by upregulation of γ-glutamylcysteine synthetase activity and resulted in reduced oxidative stress [52]. In contrary, the NO inhibitory property of *A. paniculata* was associated with its bioactive diterpenoid andrographolide, which caused the reduction of inducible NO synthase (iNOS) mRNA and protein expression [53], [54].

*A. paniculata* is reported to suppress growth of different human cancer cells, including Jurkat, PC-3, Colon 205, and HepG2 cells. Extracts of *A. paniculata* aerial parts were found to induce cell cycle arrest and mitochondrial-dependent apoptosis in human acute myeloid leukemia cells [22], [55]. The current study reports that three isolated fractions of ELAP inhibit HepG2 proliferation via mitochondrial-dependent apoptosis. The IC_50_ values of isolated fractions in HepG2 cells were estimated as 17 to 22 µg/mL, which is comparable to findings in previous studies [56], [57]. The significant LDH release, which is a marker of irreversible cell injury, also confirmed the cytotoxic effect of CF, HF, and BF on HepG2 cells. Yen et al. [58] have found that andrographolide markedly inhibits cerebral endothelial cell growth. ROS have been reported to initiate apoptotic signaling [59]. We show excessive ROS formation in HepG2 cells after treatment with CF and HF. Andrographolide is the main active compound in *A. paniculata* that triggers ROS formation in lymphoma cells and induces apoptosis via mitochondrial-mediated pathways [60]. Our experiment showed that BF did not induce significant ROS generation in HepG2 cells. CF, HF, and BF activated caspase-9 and -3/7, suggesting the induction of apoptosis via intrinsic pathways. Previous studies also showed the induction of mitochondrial-mediated apoptosis by *A. paniculata* and andrographolide on lymphoma and human leukemia HL-60 cells [56], [60]. Activation of caspase-8 by CF, HF, and BF suggests that extrinsic pathways induce apoptosis. Andrographolide from *A. paniculata* has been shown to induce cell death and cell cycle arrest at G2/M phase in HepG2 cells [57]. Andrographolide also significantly enhances tumor necrosis factor–related apoptosis-inducing ligand (TRAIL)-induced apoptosis in various human cancer cell lines [42]. Concurrent activation of both extrinsic and intrinsic apoptotic pathways by plant-mediated constituents such as curcumin has been shown previously [61]. In the present study, significant cytotoxic effect of three different isolated fractions suggested the presence of more than one active compound (andrographolide) against HepG2 cells, which remains to be determined.

## Conclusion

Based on the results from both *in vivo* and *in vitro* studies, 500 mg/kg of ELAP significantly protected against TAA-induced liver damage. The acute toxicity study demonstrated that rats treated with ELAP at 2500 mg/kg did not show signs of toxic damage. However, treatment using ELAP reduced the liver weight to almost normal. Our histopathology finding and Masson’s Trichrome staining showed the inhibitory effect of treatment with ELAP, which may be due to its ability to inhibit hepatocyte proliferation, as indicated by PCNA staining. ELAP significantly elevated the concentration of serum CAT and SOD, while it significantly decreased hepatic MDA and NO compared to the TAA control group. The pathological increases in serum levels of liver biomarkers caused by TAA toxicity were restored significantly upon ELAP treatment. ELAP fractions were free of toxic effects on WRL-68 cells, but inhibited HepG2 cell proliferation in both a dose- dependent manner. Furthermore, treatment of HepG2 cells with ELAP fractions significantly increased LDH release and ROS generation in a dose-dependent manner. A multiparameter cytotoxicity assay showed a dose-dependent reduction of MMP, a significant increase in cell membrane permeability, and a concentration-dependent increase in cytochrome *c* release in HepG2 cells treated with different concentrations of ELAP fractions. Marked dose-dependent increases in caspase-9 and -3/7 activity and a gradual increase in caspase-8 activity were detected in the treated HepG2 cells. ELAP fractions activated downstream caspase molecules and consequently triggered apoptosis in HepG2 cells that could be mediated via the intrinsic, mitochondrial-caspase-9 pathway and the extrinsic, death receptor-linked caspase-8 pathway. ELAP has thus been demonstrated to accelerate the recovery of TAA-induced liver damage and ELAP fraction possesses a significant cytotoxic effect in HepG2 cells, suggesting the involvement of several active compounds towards cytotoxic effects against HepG2 cells.

## Supporting Information

Figure S1 **Effect of ELAP on liver and kidney histology in acute toxicity study.** Histological sections of liver (A, B) and kidney (C, D) from rat treated with vehicle (10% Tween-20; A, C) or ELAP (2500 mg/kg; B, D). H&E staining demonstrates the normal structural appearance of liver and kidney parenchyma.
(TIF)

Table S1 **Effect of ELAP on liver function biochemical parameters in acute toxicity study.**
(DOCX)

Table S2 **Effect of ELAP on renal function biochemical parameters in acute toxicity study.**
(DOCX)
