# Supplementary figures and images for: Andrographis paniculata Leaf Extract Prevents Thioacetamide-Induced Liver Cirrhosis in Rats
Source: PLoS One. 2014 Oct 3;9(10):e109424. doi: 10.1371/journal.pone.0109424 (PMC4184875; doi:10.1371/journal.pone.0109424)

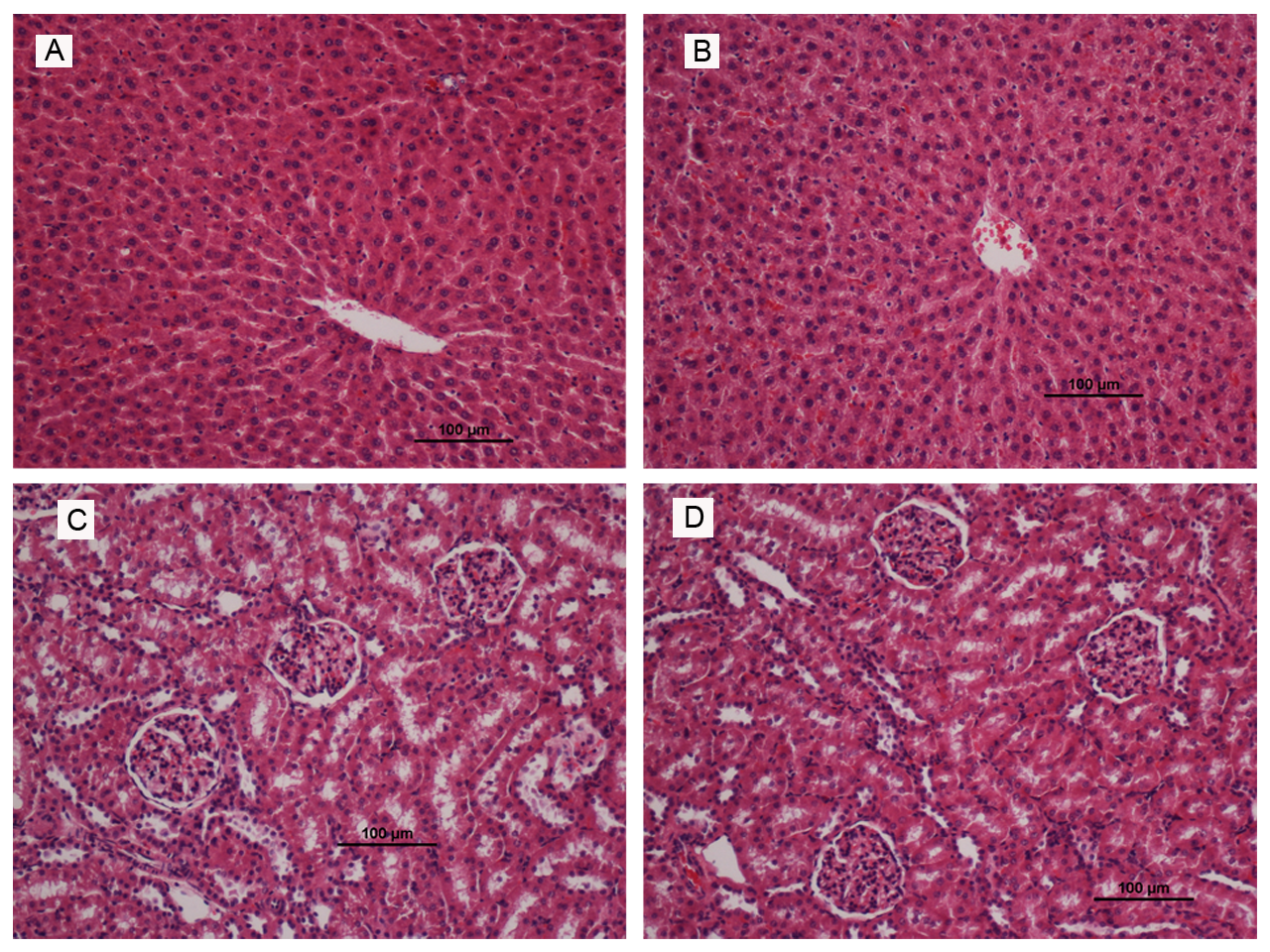

Supplement: Figure S1 — Effect of ELAP on liver and kidney histology in acute toxicity study. Histological sections of liver (A, B) and kidney (C, D) from rat treated with vehicle (10% Tween-20; A, C) or ELAP (2500 mg/kg; B, D). H&E staining demonstrates the normal structural appearance of liver and kidney parenchyma. (TIF) [file pone.0109424.s001.tif]
