# Supplementary material for: Andrographis paniculata Leaf Extract Prevents Thioacetamide-Induced Liver Cirrhosis in Rats
Source: PLoS One. 2014 Oct 3;9(10):e109424. doi: 10.1371/journal.pone.0109424 (PMC4184875; doi:10.1371/journal.pone.0109424)
Supplement: Table S1 — Effect of ELAP on liver function biochemical parameters in acute toxicity study. (DOCX) [file pone.0109424.s002.docx]

**Acute toxicity test**

**Table S1 Effect of ELAP on liver function biochemical parameters in acute toxicity study.**

| **Dose** | **Total protein (g/L)** | **Albumin (g/L)** | **Globulin (g/L)** | **TB (µmol/L)** | **CB**  **(µmol/L)** | **ALP**  **(IU/L)** | **ALT**  **(IU/L)** | **AST**  **(IU/L)** | **GGT**  **(IU/L)** |
| --- | --- | --- | --- | --- | --- | --- | --- | --- | --- |
| **Vehicle**  **(10% Tween 20)** | 71.13 + 1.46 | 11.68 + 0.53 | 58.95 + 1.35 | 1.96 + 0.18 | 0.89 + 0.17 | 134.25 + 10.29 | 53.35 + 3.53 | 153.94 + 7.15 | 4.96 + 0.98 |
| **ELAP**  **2500mg/kg** | 70.78 + 1.50 | 11.53 + 0.34 | 59.52 + 1.15 | 2.07 + 0.15 | 1.00 + 0.00 | 133.61 + 8.77 | 52.88 + 307 | 155.11 + 6.71 | 5.05 + 1.04 |

Values expressed as mean ± S.E.M. There are no significant differences between groups. Significant value at *p<0.05*
