# Supplementary material for: Andrographis paniculata Leaf Extract Prevents Thioacetamide-Induced Liver Cirrhosis in Rats
Source: PLoS One. 2014 Oct 3;9(10):e109424. doi: 10.1371/journal.pone.0109424 (PMC4184875; doi:10.1371/journal.pone.0109424)
Supplement: Table S2 — Effect of ELAP on renal function biochemical parameters in acute toxicity study. (DOCX) [file pone.0109424.s003.docx]

**Acute toxicity test**

**Table S2 Effect of ELAP on renal function biochemical parameters in acute toxicity study.**

| **Dose** | **Sodium**  **(mmol/L)** | **Pottasium**  **(mmol/L)** | **Chloride**  **(mmol/L)** | **CO_2_**  **(mmol/L)** | **Anion gap**  **(mmol/L)** | **Urea**  **(mmol/L)** | **Creatinine**  **(µmol/L)** |
| --- | --- | --- | --- | --- | --- | --- | --- |
| **Vehicle**  **(10% Tween 20)** | 138.19 + 0.41 | 5.13 + 0.19 | 104.17 + 0.18 | 23.25 + 0.86 | 18.14 + 0.95 | 5.33 ± 0.451 | 50.22 ± 1.39 |
| **ELAP**  **2500mg/kg** | 137.33 + 0.44 | 5.26 + 0.16 | 103.75 + 1.25 | 22.62 + 0.52 | 18.47 + 1.15 | 4.91 + 0.48 | 48.88 + 0.851 |

Values expressed as mean ± S.E.M. There are no significant differences between groups. Significant value at *p<0.05*

TB: Total bilirubin; CB: Conjugated bilirubin; ALP: Alkaline phosphatase; ALT: Alanine aminotransferase; AST: Aspartate aminotransferase;GGT: G-Glutamyl Transferase .
